# Earliest evidence for equid bit wear in the ancient Near East: The "ass" from Early Bronze Age Tell eṣ-Ṣâfi/Gath, Israel

**DOI:** 10.1371/journal.pone.0196335

**Published:** 2018-05-16

**Authors:** Haskel J. Greenfield, Itzhaq Shai, Tina L. Greenfield, Elizabeth R. Arnold, Annie Brown, Adi Eliyahu, Aren M. Maeir

**Affiliations:** 1 University of Manitoba, Department of Anthropology, Judaic Studies Program and St. Paul’s College, Winnipeg, Manitoba, Canada; 2 Ariel University, Israel Heritage Department and the Department of Land of Israel Studies and Archaeology, Ariel, Israel; 3 University of Saskatchewan, Department of Religion and Culture, St. Thomas More College, Saskatoon, Saskatchewan, Canada; 4 Grand Valley State University, Department of Anthropology, Allendale, Michigan, United States of America; 5 University of Manitoba, Department of Anthropology and St. Paul’s College, Winnipeg, Manitoba, Canada; 6 Ariel University, The Institute of Archaeology and the Department of Chemical Sciences, Ariel, Israel; 7 Bar-Ilan University, Martin (Szusz) Department of Land of Israel Studies and Archaeology, Ramat Gan, Israel; New York State Museum, UNITED STATES

## Abstract

Analysis of a sacrificed and interred domestic donkey from an Early Bronze Age (EB) IIIB (c. 2800–2600 BCE) domestic residential neighborhood at Tell eṣ-Ṣâfi/Gath, Israel, indicate the presence of bit wear on the Lower Premolar 2 (LPM2). This is the earliest evidence for the use of a bit among early domestic equids, and in particular donkeys, in the Near East. The mesial enamel surfaces on both the right and left LPM2 of the particular donkey in question are slightly worn in a fashion that suggests that a dental bit (metal, bone, wood, etc.) was used to control the animal. Given the secure chronological context of the burial (beneath the floor of an EB IIIB house), it is suggested that this animal provides the earliest evidence for the use of a bit on an early domestic equid from the Near East.

## Introduction

Zooarchaeological analyses from Tell eṣ-Ṣâfi/Gath provide the earliest well-dated evidence for the use of a bit in domestic equids in the ancient Near East, particularly for donkeys. Bit use on donkeys appeared in the early 3^rd^ millennium BCE in the southern Levant—the acronym BCE (Before Common/Christian Era) is most appropriate for our region of study. Analysis of an excavated completely articulated domestic *Equus asinus* skeleton from the Early Bronze Age III (EBIII) (ca. 2800–2600 BCE) deposits at Tell eṣ-Ṣâfi/Gath, Israel demonstrate that donkeys were being ridden or managed with the use of a dental bit. Based on the dental data from this animal, we suggest that domestic donkeys were being ridden and controlled with bits during the early 3^rd^ millennium BCE in the southern Levant, very soon after they were domesticated in the late 4^th^ millennium in northeast Africa [1–3].

## Donkeys in the ancient Near East

The introduction of the domestic donkey to the Near East at the end of the 4^th^ and beginning of the early 3^rd^ millennium BCE dramatically changed the nature of transportation of people and goods in early complex societies. Donkeys enhanced the exchange of transport of goods by making it easier and cheaper to transport commodities over both long and short distances, which resulted in enhanced intra- and inter-regional exchange of good and the movement of people [4–7]. Other than cattle, these “beasts of burden” allowed a flourishing of long-distance exchange networks that connected the ancient Near East to central and southern Asia to Egypt, Anatolia, and the Mediterranean coastal ports. This connectivity linked all of these regions into an interaction zone (in which both goods are traded and ideas are spread) across the landscape. Donkeys continue to be used mainly as pack animals and for labor (for ploughing, to pull water from wells, etc.) across much of the developing world (e.g. sub-Saharan Africa) [8, 9].

In Mesopotamia, there are both textual and iconographic evidence that donkeys and/or onagers (hemiones or related cross-breeds) were used for plowing and as draft animals by the early 3^rd^ millennium BCE [10]. The most famous depictions are on the “Standard of Ur” and the “Vulture Stele,” where they have been identified as pulling wagons, through the use of nose rings [10–19]. Donkeys are identifiable in bas-reliefs in tombs where they are depicted as being used for threshing and transport of goods in Egypt from the Old Kingdom onwards [20–25].

Based on zoomorphic figurines of domestic equids that appear during the latter half of the 4^th^ millennium BCE in the Near East (Early Bronze Age/EB I in the southern Levant; and end of the Chalcolithic in Mesopotamia), donkeys were at this point clearly used as beasts of burden [16, 26–31]. Various depictions show donkeys carrying a variety of objects (jars, containers) (see for example [7]) and/or encumbered with saddles or harnesses, although is not clear if they were also ridden [32]. Interestingly, laden animal figurines are not commonly represented after the Early Bronze Age and as such may very well reflect the new significance of the donkey in the EB, while not portraying the then established everyday role of the donkey in transportation and commerce in post-EB time periods.

The importance of the donkey in the development and maintenance of interregional trade is evident from Old Kingdom Egyptian texts. During this period, donkeys were already being raised on a large scale to meet the increased demand for the transport of goods between Egypt, and the surrounding regions: Sinai and the Levantine Coast to the east, the western desert, and Nubia to the south [25]. For example, a land owner from Dynasty 4 possesses over 760 donkeys. *Herkhug* (the caravan master of Pharaoh *Meren-Re* from Dynasty 6) returns with 300 donkeys carrying incense, ebony, and grain from Nubia. Later, during the Middle Kingdom (Dynasty 11), another official (*Sinhue*) leads donkey caravans that are transporting goods between Egypt and the southern Levant (in the “Story of the Eloquent Peasant”) [5, 21, 23, 33–35]. Recent isotopic evidence from animals at Safi provides direct evidence of donkeys moving between Egypt and Israel during the Early Bronze Age [36].

## Antiquity of the bit in the Near East

In contrast to what is known about the use of donkeys for transportation, relatively little is known about their use for riding during this early period [37]. Riding is possible, but fast riding is difficult without some kind of bridle with reins to grasp. Thus, the development of the bit becomes an essential part of the mechanism to control and ride an equid, whether horse, donkey or otherwise [38–41]. While some have tried to argue based on cave art for the presence of bridles (including cheek straps and potentially bits) on equids as far back as the Upper Palaeolithic [42, 43], this perspective has not been accepted [44, 45]. Instead, the weight of the evidence for bridles points toward the Eneolithic and Bronze Age of Kazakhstan and Russia, c. 3500 BCE for horses, not donkeys [38, 40, 46–50]. But, horses are not the earliest domestic equids to appear in the Near East. This role is reserved for the ass/donkey [20, 32, 51].

The literature is rife with ambiguous terminology that fails to distinguish between halters and bridles. A halter is used for leading or tethering, while a bridle allows for much finer control of the animal, particularly for riding or steering. Both are part of the headgear used to control an animal (usually an equid), with which reins may be attached. A halter can be a rope or strap with a noose placed around the back of the head, across the cheeks and around the nose of the animal [52]. While both a halter and bridle use cheek straps that encircle the mouth, a bridle also includes a bit that extends through the mouth and leans up against the lower premolar 2 (LPM2) teeth.

The importance of the bit is that it impacts the way in which domestic transport animals can be used. A bit helps with the training, conditioning, redirection, and control of animals, particularly when not used in a group situation. This is especially relevant in situations where the animal is being ridden and absolute control of the animal is essential [48, 52, 53]. A bit is not necessary if the animal is simply being led around. For example, rather than being bitted, caravan animals are often tied one behind the other if there are not sufficient drivers. They will follow each other without being tethered since they are behaviorally social animals. This scenario makes sense when one keeps in mind the iconography of early equids in the Near East. Images portrayed are of donkeys being controlled by nose rings or bands while they pull wagons or carry people and goods [19]. But, as has been observed with modern equids, nose rings and bands are a very poor means for controlling equids, in particular donkeys. Nose bands slip around the nose when the reins are pulled [52]. Similarly, one cannot ride a donkey or have a donkey pulled a wagon by pulling on a nose ring since any side to side or back and forth movement will pull the ring out of the soft tissue of the nose. Nose rings only effectively function as control when used from the front of the animal. As is evident to anyone who has experienced trying to control a large animal with a nose ring, they can be used only to have an animal follow one behind the other or to tether an animal to a fixed placed so that it cannot wander. They cannot be used effectively as a bridle substitute for a rider or a driver [52].

Recently, Milevski [5] suggested that there was an evolution in the use of the ass during the EBA of the southern Levant based on changes in the appearance of figurines since the earliest figurines only depict animals carrying goods as ‘beasts of burden’ (during the EB I-II). Chronologically, the late Chalcolithic of the ancient Near East (Mesopotamia, Syria, etc.) is synchronous with the EB I of the southern Levant. It is only in the subsequent period that figurines show that they were also being ridden (EB II-III). Given this change in function, we expect to find osteological and/or artefactual evidence of donkeys being ridden beginning in this period.

The appearance or identification of bridles and bits in the archaeological record has often been tied to discussions concerning whether early equids (horses, donkeys, onager, etc.) were domesticated. Archaeologists and zoologists working on material from Europe and the Near East have extensively studied the disparate iconographic, textual and artefactual remains to determine when equids were first ridden, e.g. [17, 20, 37, 40, 41, 44, 48, 50, 52, 54, 55, 56]. Pieces of worked bone and antler have long been suggested as potential bridle and cheek pieces in Europe and Eurasian steppes from the late third, but more commonly early second millennium BCE sites [39, 47, 52, 54, 57–59]. However, there is no agreement in the literature that these objects are actually cheek pieces since they have never been found *in situ* on an equid cranium and rarely found in association with horse burials [60].

In graves dated from the Akkadian period and toward the end of the EB (late in 3^rd^ mill. BCE) at Tell Brak in northern Mesopotamia (modern Syria), it has been suggested that the differential wear visible on anterior face of the LPM2 of donkey teeth was a function of bit wear [11, 14, 15, 61]. In addition, there is a ceramic plaque recovered from Akkadian deposits at Kish that depict an ass or onager being ridden [17, 19, 62].

The earliest unambiguous evidence for bridles and bits in equids in the Near East appear only in the Middle Bronze Age [52, 62, 63], and horses become common only in cuneiform texts and the archaeological record after the turn of the second millennium BC [44]. For example, at the Middle Bronze Age site of Tel Haror, a metal bit was found associated with a donkey burial [63].

Beginning in the Middle Bronze Age, there is a variety of sources that demonstrate that asses were being ridden. In fact, they seem to be the preferred animal ridden for elites in the Early and Middle Bronze Age of Mesopotamia. The earliest clear association of asses being ridden by elites comes from the Old Babylonian period (MBA, 18^th^ century BCE—the Kings of Mari, Syria) [64]. Similarly, by the beginning of the Middle Kingdom of Egypt, various texts and iconographic images (e.g. the stela of Serabit el-Khadem) from Egypt and petroglyphs from southern Sinai unambiguously depict and/or describe elites riding asses [5, 65, 66]. The later biblical narrative depicts donkeys carrying the biblical Patriarchs (Abraham), various leaders (such as Saul before he became king), prophets, and judges of Israel [16, 67, 68].

Horses became the standard royal riding animal during the Late Bronze and Iron Ages as they became more prevalent. In later periods, donkeys became associated with humility and the lower classes, and leaders emanating from it (e.g. Jesus).

## Identifying bit wear

In the absence of clear bridles and metal bits, bit wear can be (and has been) used as a proxy diagnostic. The bit normally will sit on the tongue and gums in the diastema between the premolars and the incisors which will result in morphological pathologies (e.g. the wearing down of the tooth surface due to the rubbing) on the mandibular (diastema) bone and dental enamel of the LMPM2 [69, 70]. Bit wear usually occurs as a result of two scenarios. First, when the rider or driver exerts pressure on the bridle or reins, the bit will slide back in the mouth until it rests against the mesial surface of both LPM2’s. The animal’s response to the presence of the bit is to use its tongue to elevate and retract it against the upper mesial surface of the LPM2 [71]. In the second scenario, both the LPM2 and UPM2 are affected. The animal responds to pressure by elevating the bit with their tongue slightly higher in the mouth where it comes into the grip of their lower and upper PM2s. In this position, the bit will not cause any pain, no matter how hard the rider pulls on the reins, but it can be chewed on (as some equids like to do). The back and forth pulling on the bridle appears to affect the lower PM2 more, in particular the superior half of the mesial face [54, 70].

Repeated grinding and/or impact by a bit will cause unnatural and differential erosion of the enamel and exposure of the dentine. Bit wear is created by both the bit grinding against the tooth enamel and by the grit caught between the bit and the teeth which will unnaturally wear the tooth’s surface down. As noted elsewhere, this is why even a soft (e.g. leather) bit can wear away at the enamel [71]. The slipping of the bit back and forth over the prow of the LPM2 creates a notable beveled edge [54, 71]. A bevelled edge is when a straight or square edge of an object has been reduced to a sloping edge. The result is that the mesial face of the enamel of the LPM2 immediately below the occlusal (chewing) juncture is pathologically modified, particularly the paraconid (or first) cusp of the tooth. This type of wear on the LPM2 occurs most commonly with hard bit types, but the patterns are present although less obvious diagnostic in softer (e.g. organic) bit. In wild and unbitted equids, the juncture of the mesial-occlusal surfaces of the LPM2 is flat and squared [41, 48, 53, 69, 72].

## The specimen—Description and archaeological context

**Fig 1:**
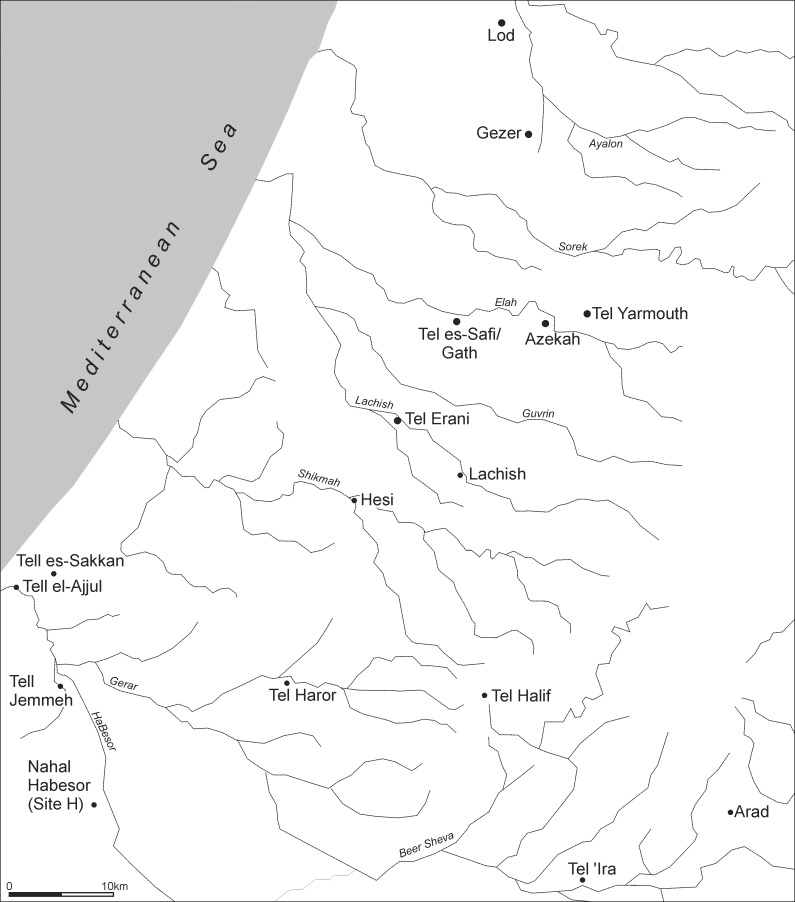
Map of region showing location of Tell eṣ-Ṣâfi/Gath and other important Early Bronze Age urban centres in the region.

Ongoing excavations at Tell eṣ-Ṣâfi/Gath have revealed a wide variety of evidence as to the significance of early domestic donkeys in the third millennium BCE. Located in the *Shephelah* (Judean foothills) of central Israel, it overlooks and dominates the coastal plain and monitors an important route between the foothills and the coast and traffic moving inland towards the mountainous interior of the country (Fig 1).

**Fig 2:**
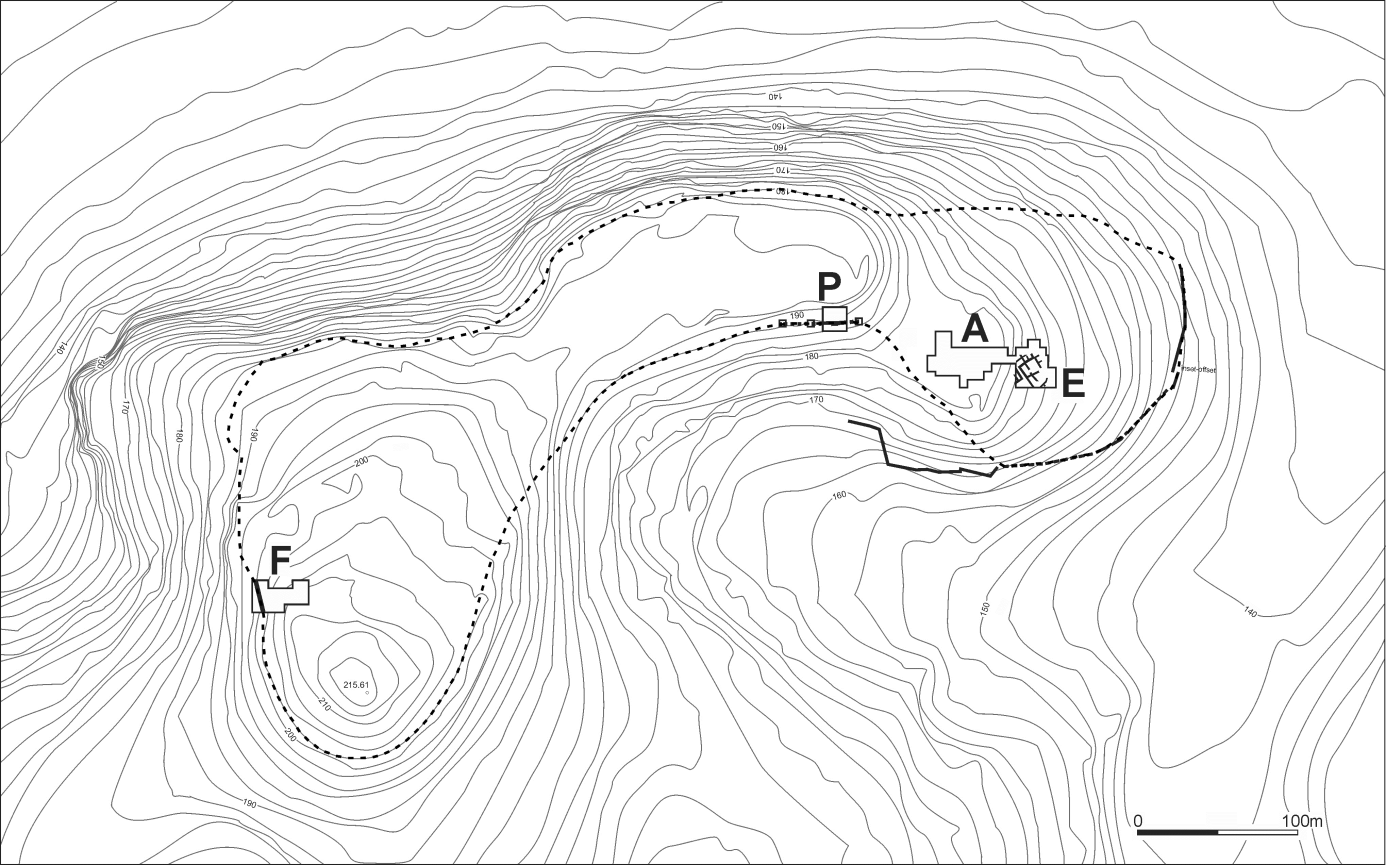
Map of Tell eṣ-Ṣâfi/Gath showing extent and excavation areas. Excavation Area E is where the specimen being discussed is found.

**Fig 3:**
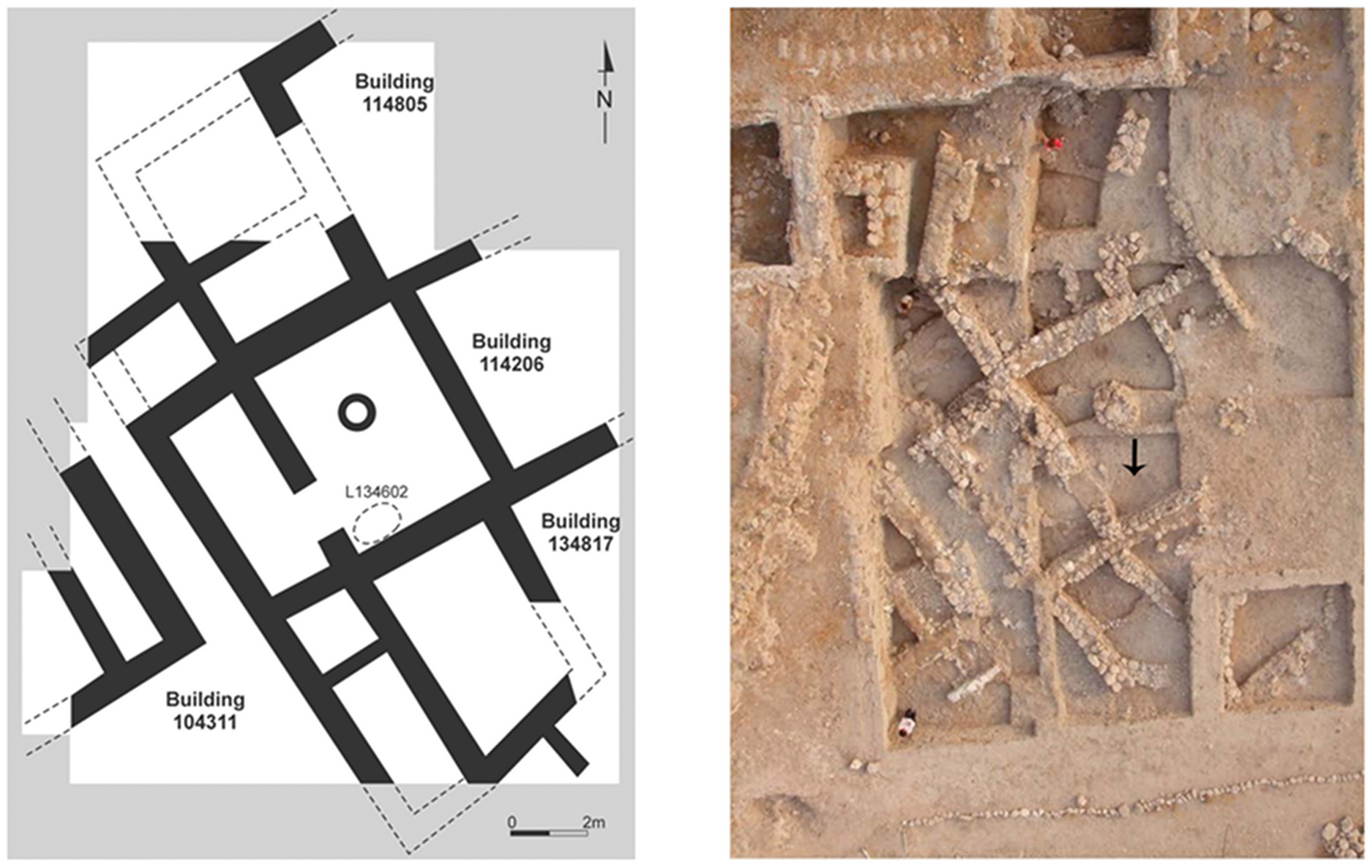
Plan and photograph of architecture from Stratum E5 in Area E at Tell eṣ-Ṣâfi/Gath and location of donkey burial. A–Plan with dotted circle that shows location of burial; B–Aerial photograph of EB architecture showing location of burial with arrow. Site north is at top of photo.

The entire site is underlain by a large and extensive EB urban center (c. 24 ha in area) that assuredly dominated the region as an independent city-state. It was densely occupied across its entire extent and surrounded by a large stone wall fortification system (Fig 2) [73]. At the eastern end of the site (Area E), excavations uncovered the remains of several private houses in what we interpret as a domestic quarter (Fig 3a and 3b). The neighborhood and its structures went through several phases of occupation dating to the EB III (Stratum E5 and E6) and II (Stratum E7) [74–76].

**Fig 4:**
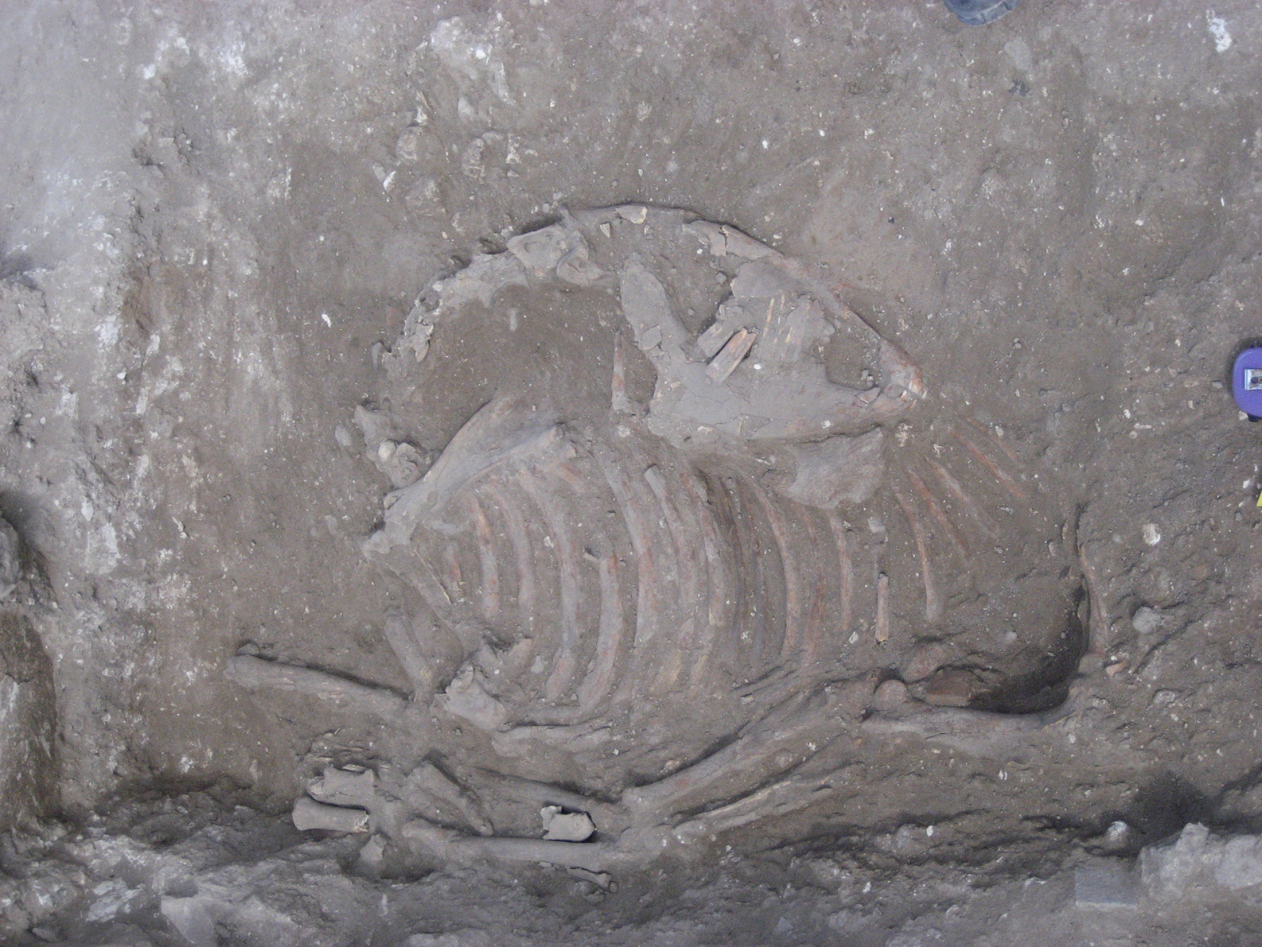
Photograph of donkey burial from the E5c Stratum of Area E at Tell eṣ-Ṣâfi/Gath in Area E as it was being uncovered; facing north. Site north is at top of photo.

The remains of a fully articulated domestic ass or donkey (*Equus asinus*) skeleton was found (Locus 134602) in a shallow pit dug into the mudbrick collapse of the house from the underlying E6 Stratum (Fig 4). The burial is sealed beneath the beaten earth floor (Locus 114502) in the courtyard (Room 144602) of a house (Building 134307), without any evidence of intrusion. All of the artefactual material in the deposits above, around, and below the burial pit belong to the later EB III. House (Building 134307) is dated to the beginning of Stratum E5c, which is at the beginning of the late EB III (or EB IIIB) occupational stratum at the site. The end of the stratum is tightly dated by radiocarbon analysis to c. 2600 BCE. The EB III, as a period, is c. 300 years long for the region in general and has been tightly dated by radiocarbon analyses to c. 2850–2500 BCE [76, 77]. Unfortunately, the absence of preserved collagen in the skeleton prevented its direct radiocarbon analysis. Yet, there is little reason to doubt its chronological integrity. As a result, the burial’s stratigraphic position suggests that it should be dated to the beginning of the E5c Stratum and probably dated to c. 2700 BCE. In addition to the secure stratigraphic and chrono-typological dating of the donkey burial, radiocarbon dating of the underlying (earlier) EB stratigraphic sequence in Area E is currently in process, but are not expected to change the dating of the burial.

Based on zooarchaeological, architectural, stratigraphic, and typo-chronological analyses of this bioarchaeological deposit, it is clear that the ass was deliberately bound, slaughtered, and buried as a foundation deposit before the late EB III (Stratum E5c) house was constructed. While there are no clear and unambiguous cut marks, the head and upper neck (C1-5) are separated from the rest of the body. They are placed on top of the right side of the upper rib cage while facing toward the rear of the skeleton. The C6 is destroyed and missing. The legs are bent in such a manner as to suggest that they were likely bound together given their anatomical position [75, 76]. The floor of the house seals the deposit.

**Fig 5:**
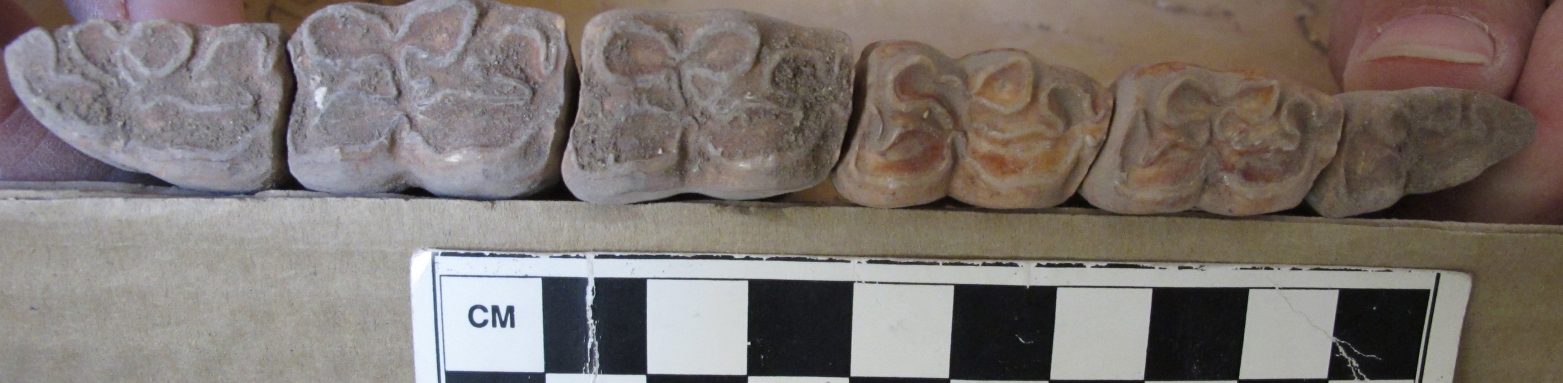
Photograph of entire lower right premolar and lower molar tooth row on the donkey skeleton from the E5c Stratum of Area E at Tell eṣ-Ṣâfi/Gath.

**Fig 6:**
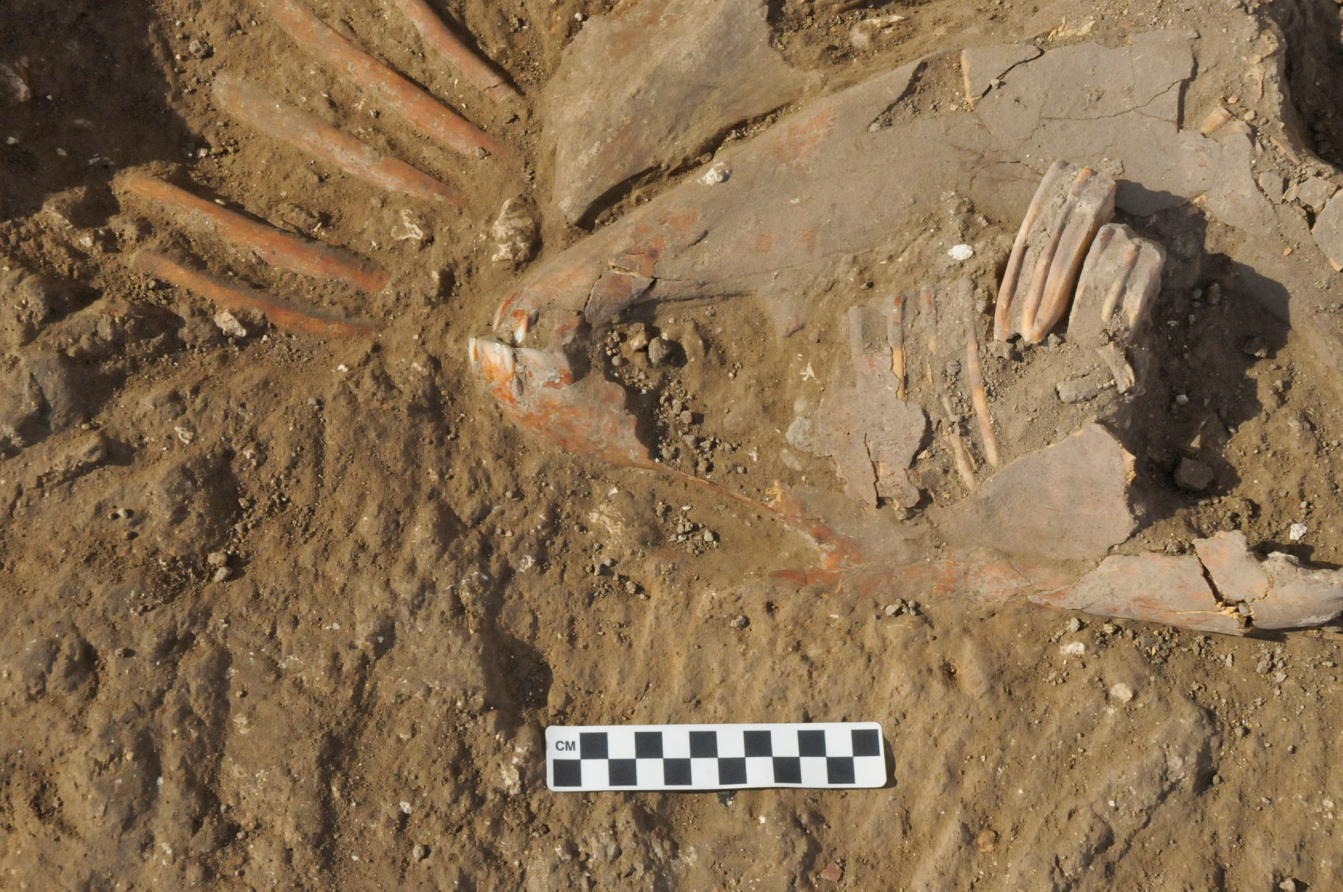
Close-up photograph of right side of donkey cranium and mandible *in situ*. It shows the absence of canines and any evidence of rubbing on the diastema of the mandible on the donkey skeleton from the E5c Stratum of Area E at Tell eṣ-Ṣâfi/Gath.

The donkey was a healthy relatively young female (4–5 years of age) based on epiphyseal fusion and tooth eruption and wear sequencing–all long bones were fully fused, while the vertebral plates at the cranial and caudal ends of most vertebrae were still unfused or partially fused [78, 79]. Further, while each adult check tooth had emerged and was in wear, the tooth wear was not advanced (Fig 5). The absence of canines (Fig 6) and the shape of the pelvis suggest that it was a female [79, 80, 81–84].

**Fig 7:**
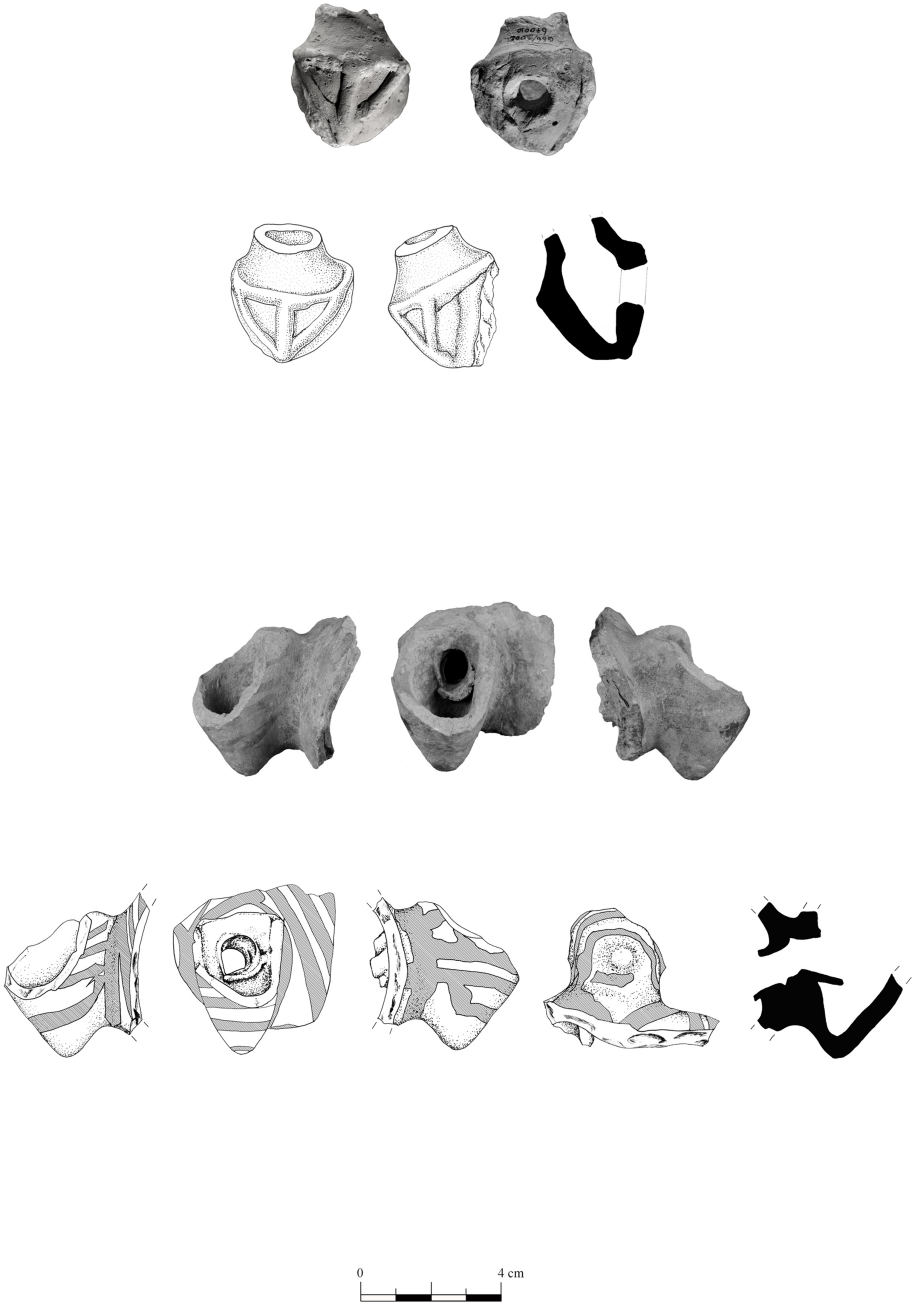
Drawing of fragments of ceramic zoomorphic vessels depicting donkeys carrying loads recovered from Tell eṣ-Ṣâfi/Gath, Area E. A. Reg. # 949072/1, Locus 94911; B. Reg. # 670010, Locus 580400. [Reprinted with permission from 7: Tafel 5].

Figurines from Tell eṣ-Ṣâfi/Gath and elsewhere in the southern Levant depict donkeys carrying large (and probably heavy) loads on either side of their body [7] (Fig 7). Our specimen was likely used as a beast of burden as there are a few minor distal limb osteological pathologies characteristic of carrying a heavy burden over uneven ground. Carrying heavy loads can create mechanical stress on joints. This is reflected in the metatarsus, which displays a depression on the proximal articular surface toward the cranial/anterior edge, stronger than expected lipping on both the proximal articular surface and both faces of the distal condyles, and greater than expected muscle attachments on the posterior face of the shaft. Two pathologies are also identified on the first phalange: a) Both the proximal articular surface and distal condyles display strong peripheral and irregular lipping, particularly toward the caudal face, instead of being smooth and gradual; b) The two articular facets on the posterior (or caudal) face for the distal sesamoids appear to be inflamed with sesamoiditis. There is also a thickening of the epiphyseal plates or facets on the articular facets of the sesamoid facets for the distal end of the first phalange. These patterns are not normal for such a young animal [7]. It is unlikely that the pathologies identified here are the result of congenital defects given that they are all located on the important joints necessary for mobility.

Isotopic analysis of the cheek (M1-3) from the Tell eṣ-Ṣâfi/Gath specimen indicate that it was born and raised in Egypt, and only came to Tell eṣ-Ṣâfi/Gath in the last few months of its life [36]. Given the contemporary Egyptian Old Kingdom historical texts which record caravans consisting of hundreds of donkeys carrying goods to Egypt, it is likely that the donkey came as part of these caravans and was sacrificed shortly after it arrived at the site.

## Analytical results

### Evidence of dental pathology from use of bridle with a bit

**Fig 8:**
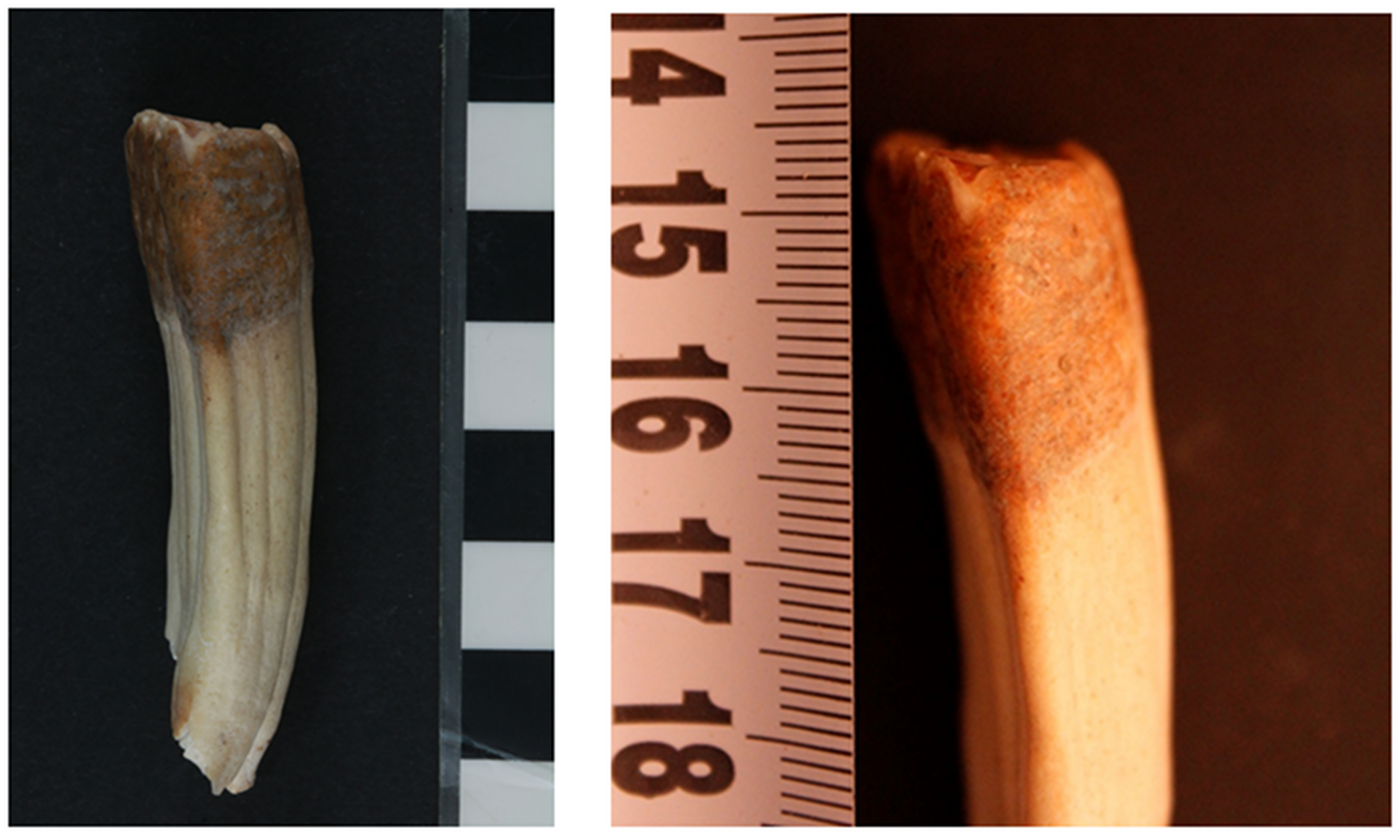
Photographs of a) full length and b) close-up of mesial/anterior face, c) full length of mesial-buccal and d) lingual views left LPM2 tooth that shows evidence of erosion or beveling of the enamel that is characteristic of bit wear on the donkey skeleton from the E5c Stratum of Area E at Tell eṣ-Ṣâfi/Gath.

There is no visible evidence of rubbing of the bridle on the superior surface of the diastema of the mandible on the bone from the Tell eṣ-Ṣâfi/Gath specimen (Fig 6). Examination of the mesial face just below the occlusal face of the left LPM2 on the Tell eṣ-Ṣâfi/Gath specimen [75] yields evidence for dental pathology that is suggestive of bit wear (Fig 8a). There is a slight unnatural and differential erosion of the enamel and exposure of the dentine on the anterior/mesial face of the LPM2 beginning at and extending to just below the occlusal surface on both LPM2s. The enamel wear along the mesial face extends downwards from the beveled edge of the occlusal face for c. 2.25mm (visible in mesial, buccal and lingual views–Fig 8b, 8c and 8d). While the length of the beveled edge is below the 3mm threshold for cultural indicators of bit wear, the threshold was established with the use of bits with extensively ridden (older) horses. It is also much higher than that found in Pleistocene fossil wild or modern domestic horses that were never subjected to a bit, where a bevel of 2 mm is very unusual and a bevel of 2.5 mm is very rare [54, 69, 71]. Also, the bit wear threshold has never been established for donkeys, which have smaller mouths and would have had smaller bits. Hence, the threshold for bevel length in horses may be too large for donkeys.

The enamel and cementum begin to be worn away starting at the mesial occlusal edge of the LPM2. The erosional surface also extends down the tooth for a few mm with decreasing depth through the enamel’s surface. The enamel is exposed at the top of the beveled surface where it meets the occlusal surface, although it is not exposed at the bottom of the beveled surface. Instead of a nearly 90-degree angle from the occlusal surface to the lingual face of the LPM2 in non-bitted equids, the beveled edge slopes at approximately a 63-degree angle downwards when measured from the occlusal surface (as 0 degrees) towards the front of the mandible. The morphology of the wear pattern (as a thin strip extending downwards along the mesial face of the LPM2 from the occlusal edge) on the Tell eṣ-Ṣâfi/Gath specimen at the mesial corner of the occlusal face is similar to the beveling described by several other authors who have examined ancient and modern domestic equid remains [69, 72, 85–88]. This pattern of uneven enamel wear suggests that a bridle bit was employed to control the animal.

**Fig 9:**
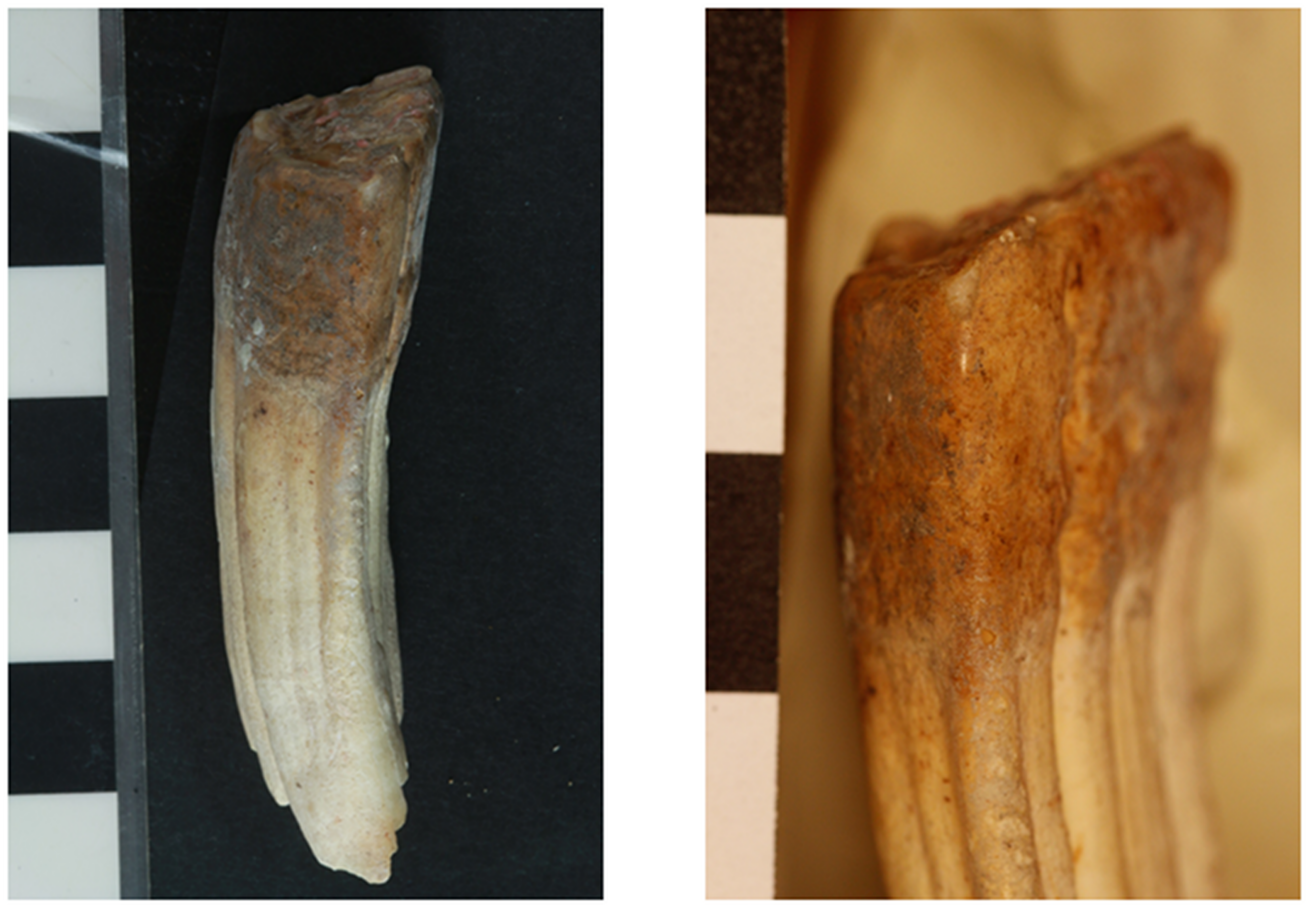
a) Full length and b) close-up photographs of mesial/anterior face of right LPM2 tooth that shows evidence of erosion of the enamel that is characteristic of bit wear on the donkey skeleton from the E5c Stratum of Area E at Tell eṣ-Ṣâfi/Gath.

**Fig 10:**
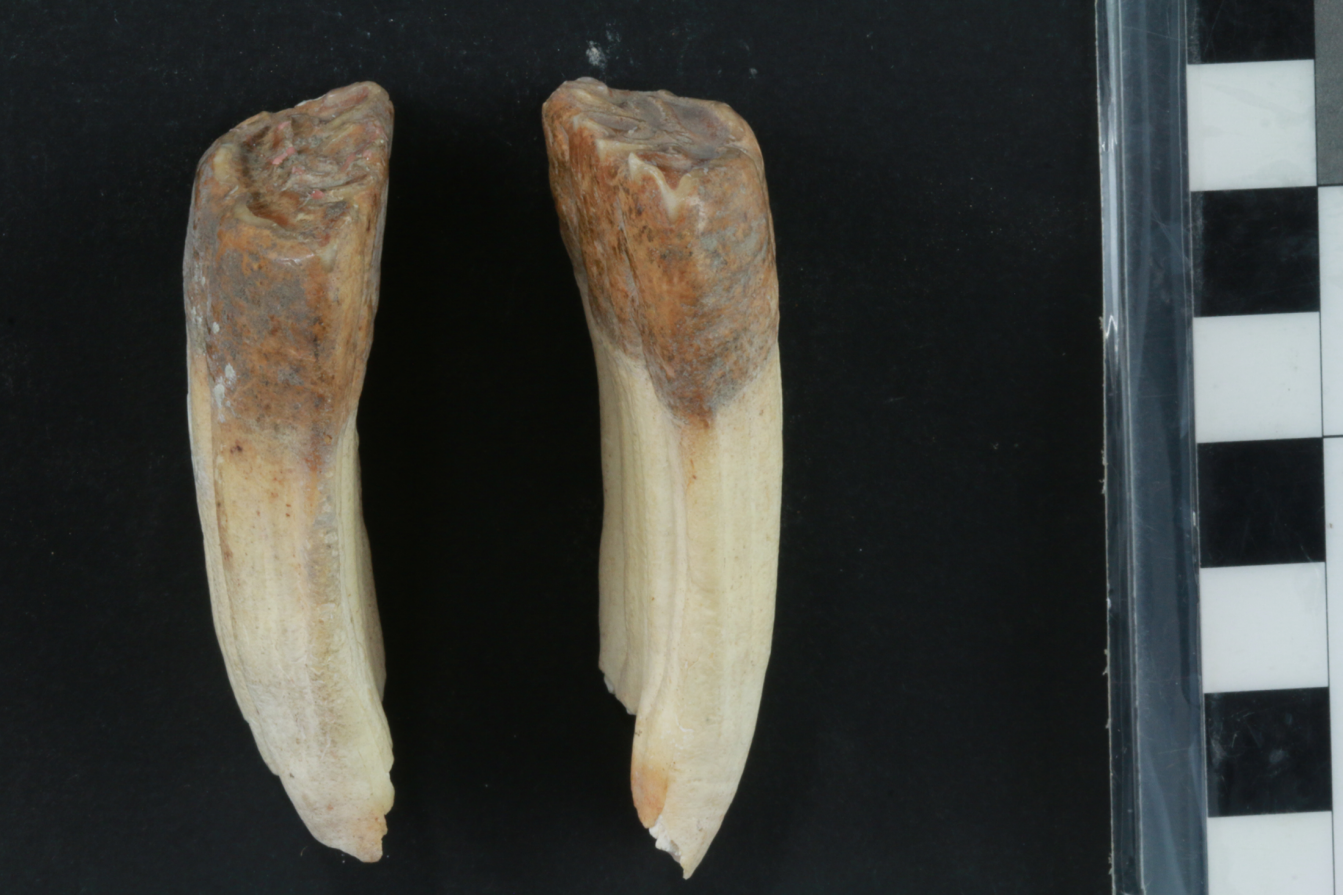
Photograph of mesial/anterior face of both left and right LPM2 teeth that shows evidence of erosion of the enamel that is characteristic of bit wear on the donkey skeleton from the E5c Stratum of Area E at Tell eṣ-Ṣâfi/Gath.

**Fig 11:**
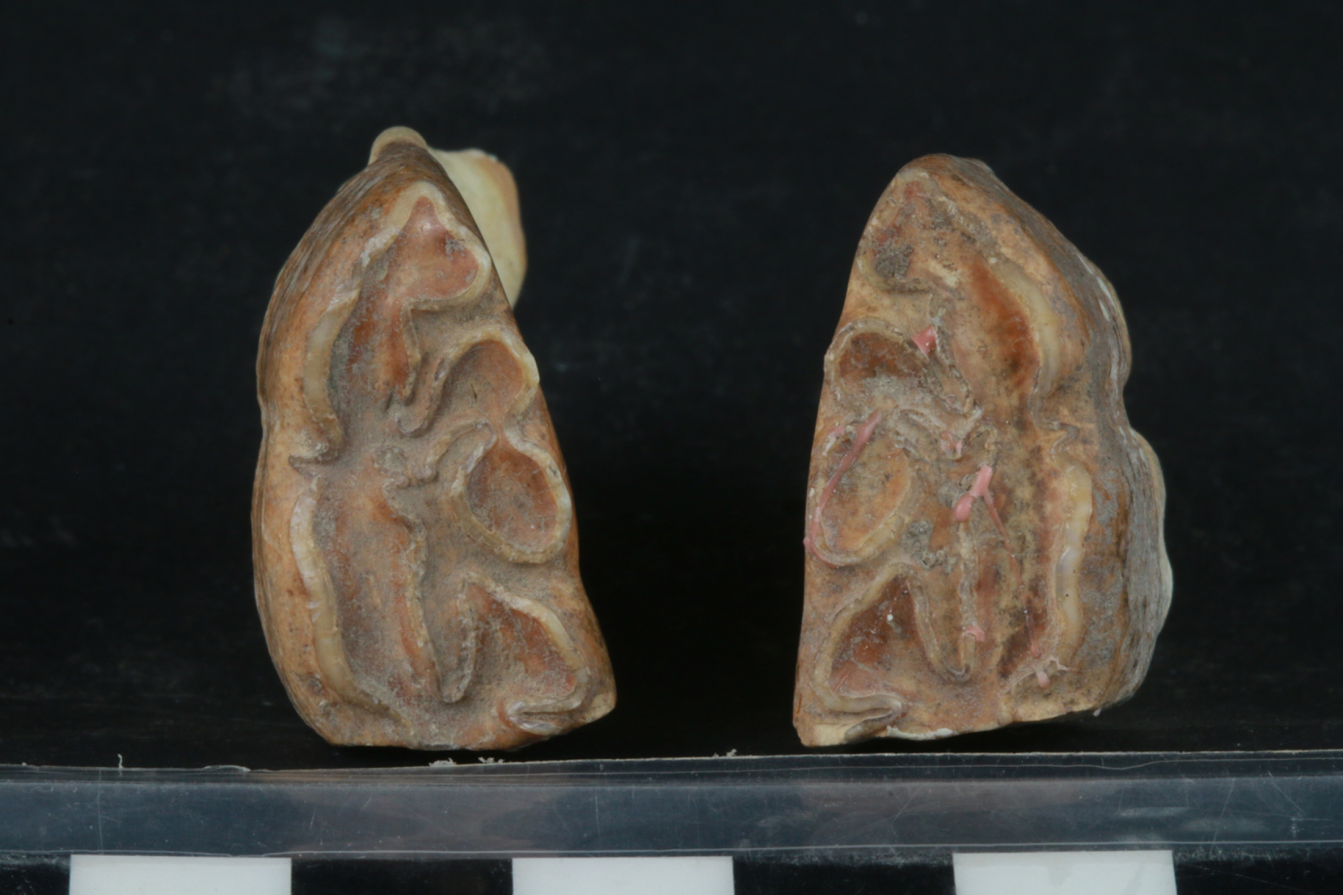
Photograph of occlusal face of both left and right LPM2 teeth that shows evidence of beveling of the enamel that is characteristic of bit wear on the donkey skeleton from the E5c Stratum of Area E at Tell eṣ-Ṣâfi/Gath.

A similar pathological pattern is visible on the right LPM2, even though it is not completely symmetrical with the left side (Fig 9a). The beveling is only slightly less on the right than left side LPM2, but it is clearly present in both (Fig 10). There is no significant perceptible difference in the morphology of the beveled edge between the left and right LPM2s (Fig 11). As a result, it is unlikely that the beveling was created by differential occlusion between left and right and between mandibular and maxillary teeth [72]. This implies that a single object was placed across the entire width of the diastema anterior to the LPM2, which is another line of support for the use of a bit as opposed to other sources of dental erosion. Further proof against the beveling being a result of malocclusion is the lack of evidence for any associated pathology on the UPM2. There is no occlusal pattern on the maxillary PM2 that might have created the beveling on the LPM2.

Bendrey [67] notes that any similar mesial enamel wear and dentine exposure on the upper teeth, or on the lingual or buccal faces, particularly in an approximately parallel-sided band may be the result of other phenomena (e.g. diet, chipping, etc.). Examination of all surfaces of all upper and lower teeth on the Tell eṣ-Ṣâfi/Gath specimen demonstrate that there is no similar pattern of wear. It is only present on the mesial surface (immediately below the occlusal zone) on both LPM2’s.

### Nature of the bit

While no metal or other type of artifacts that could be identified as a bit were found associated with the Tell eṣ-Ṣâfi/Gath specimen, striations and pitting on the surface of a tooth can also be used as a measure of differential types of bit [41, 48, 72]. On the mesial face of the tooth, metal bits create microscopic striations (from scraping up and down) and pits (from impact or material getting caught between the bit and tooth, e.g. grit). The absence of such pits and striations on the Tell eṣ-Ṣâfi/Gath specimen when examined under microscopic conditions suggests that the bit may have been of a softer nature [41, 48]. Deep striations would be a result of a hard bit. Pitting can occur under both conditions, but are more likely with a hard bit that would impact the teeth much more dramatically when as the reins are pulled. Experiments have demonstrated that even a soft bit will leave some evidence of bit wear on the LPM2 [48, 69]. While Levine [60] dismisses such results as inconclusive by arguing that none of the soft-bitted horses showed a significant bevel after 150 hours of riding, this ignores without justification the weight of the evidence that is presented in the experimental studies.

Elsewhere, Scanning Electron Microscope analysis has detected the presence of iron associated with bit-wear on domestic horse teeth [89]. In the EB, we would expect metal bits to be copper-based. However, XRF analysis did not yield any evidence of metal residue (i.e. copper/bronze) on the Tell eṣ-Ṣâfi/Gath specimen. It is unlikely that such rare metals would have been used for such mundane items. As a result, we suggest that the bit may have been made of relatively soft material (e.g. bone, wood, hard rope, etc.). A soft bit should not be discounted as recent research on such bits are coming into consideration [52]. For example, Bendrey recently suggested that a LB/EIA horse specimen from Runnymede Bridge (UK) may exhibit evidence for tooth enamel damage with a soft bit. But, as he notes, “Very few specimens have been examined from prior to the first millennium BC and use of an organic mouthpiece could significantly predate this evidence” [47, 72].

## Interpretations and discussion

The extent of the enamel wear (and exposure of the dentine) in the Tell eṣ-Ṣâfi/Gath specimen is similar to, but much less extensive than is evidenced in other studies of bit wear, probably the result of its youth. Most of the other published materials were based on older animals where the bit wear was much more advanced (e.g. [38, 69, 72, 88]). For example, Bendrey [72] suggests that an erosional zone in the enamel along the mesial face of 5 mm or less is probably a result of chipping, age, or a some dietary phenomenon, and should not be used as evidence for bit wear. However, this guideline does not take into account situations where the individual is young and whether bits were made of soft materials [48].

The Tell eṣ-Ṣâfi/Gath donkey specimen was approximately 4–5 years of age when it died, based on the epiphyseal fusion data described above. In particular, the lack of fusion of the vertebral plates suggested that it was a very young adult [75] and hence died too young for there to have been extensive damage to the teeth from a bit. It is likely that the wear is limited to a shallow depth (2.25mm) immediately below (distal to) the occlusal surface because the animal wore a bit for a very short period of time, due to its young age [48].

It has been suggested that differential chipping and the presence of microscopic spalls and/or cracks around the occlusal edge, particularly on the mesial surface, could be mistaken for bit wear [69, 72]. This pattern of malocclusion appears on the occlusal surface of many cheek teeth, rather than being limited to the anterior face of the LPM2. In the Tell eṣ-Ṣâfi/Gath specimen, there is no evidence for any of these patterns. Hence, it is unlikely that the beveling pattern described for the anterior face of the LPM2 had other causes, such as diet or feeding patterns.

Similarly, crib biting would not affect the LPM2 in this manner, as it is more focused on the incisors [42, 45, 90]. It is also unlikely that the beveling on the LPM2 was purposely caused by rasping, as suggested by Payne [91], since there is no evidence for the purposeful rounding, microscopic wear pattern and grooves associated with such rasping and the observed beveling is limited to the most superior segment of the surface which would be the first location to be worn down under the influence of a bit [72, 92].

### Conclusions

While there are no depictions of donkeys with bits from the EB of the Near East or elsewhere, the dental pathologies described in this paper suggest that the donkey excavated beneath the house floor in a domestic residential neighborhood at Tell eṣ-Ṣâfi/Gath dating to the middle of the EB III (c. 2700 BCE) may have been controlled with a bit placed in its mouth across the diastema. Given the absence of metal objects in the burial and the lack of evidence for metal residue on the teeth, the use of an organic bit (e.g. bone, wood, hard rope, etc.) is considered likely. The bit moved around the mouth sufficiently to bevel the mesial edge of the LPM2 in a manner commonly seen in equids who use bits and not seen in unbitted animals. Regular tooth wear is characterized by an even wear surface that is flatter and less polished. In contrast, only normal tooth wear patterns are evident in the UPM2s and all the other teeth in the mouth. Only the LPM2’s exhibits this unique type of wear. This pathology represents the earliest evidence for the use of a bit in the ancient Near East, and substantiates the proposal that the Tell Brak donkeys also exhibited bit wear [14, 15, 61].

These finds suggest that bit use on donkeys was already present in the early to mid-3^rd^ millennium BCE, long before the appearance of horses in the ancient Near East. Thus, the appearance of bit use in donkeys in the ancient Near East is not connected to appearance of the horse, contrary to previous suggestions (as already noted by [62]). As such, the impact of the domestic donkey on the cultures of this region and the evolution of early complex societies cannot be underestimated. As with plant and animal domestication, the use of donkeys created a surplus of human labor that allowed for the easy transport of people and goods across the entire Near East. These changes continue to permeate the economic, social, and political aspects of even modern life in many third world countries [3, 8, 9, 93, 94].

## Materials and methods

All necessary permits were obtained for the described study, which complied with all relevant regulations. Excavations permits were issued to Aren Maeir by the Israel Antiquities Authority (Permit # G-56/2008) and the Israel Nature and Parks Authority. Permits for temporary export of specimens from Israel were issued to Aren Maeir and Haskel Greenfield by the Israel Antiquities (Permit #13840/2012). All specimens are curated and available for analysis at Bar-Ilan University. Contact Aren Maeir to examine original specimens.

As with other reported on donkey specimens from the region, all non-dental osteological remains of the skeleton were fragile and badly preserved. They were intact upon discovery, but began to disintegrate upon exposure. It was impossible to preserve them intact when lifted during excavation.

During excavation, the surrounding sediment was removed in order to expose the entire skeleton. During excavation, all intact portions of bones were measured and all bones (large and small) documented with field drawings and photographs.

The specimen was identified as a domestic donkey first by Haskel and Tina Greenfield, and later confirmed by Hadas Motro. Identification is based on the enamel fold patterns on the occlusal surface of the cheek teeth and measurements of the crown heights [95–99]. The age of the specimen is based on a combination of epiphyseal fusion and tooth eruption [78, 79].

All bones were examined under a hand-held magnifying lens to determine if there was any evidence of cultural and/or natural bone surface modifications (such as butchering, burning, gnawing, tool or ornamentation, weathering, etc.) [100–102]. None were identified as being culturally or naturally modified. Pathological bone alteration was identified on some of the distal limb bones and reported upon elsewhere [7]. All relevant dental measurements are provided in Table 1.

**Table 1:** Mandibular dental measurements for Equus asinus specimen from Locus 114506, Basket 1145054.

*Element*	Length (cm*)	Breadth (cm)*	*Crown height lingual*	*Crown height buccal*
Length of premolar row	7.5			
Length of molar row	8			
Length and breadth of LPM2	2.72	1.49	4.75	4.9
Length and bread of LPM3	2.71	1.83	7.7	7.71
Length and breadth of LPM4	2.68	1.81	6.32	6.6
Length and breadth of LM1	2.5	1.6	6.8	6.1
Length and breadth of LM2	2.32	1.44	6.7	7.2
Length and breadth of LM3	2.48	1.13	6.6	6.78
Canines	Not present			
Bit wear	Present only on LPM2—polished and wear on superior edge of mesial/anterior face			

* cf. [100]
