# Correction: Earliest evidence for equid bit wear in the ancient Near East: The "ass" from Early Bronze Age Tell eṣ-Ṣâfi/Gath, Israel

**DOI:** 10.1371/journal.pone.0202382

**Published:** 2018-08-09

**Authors:** Haskel J. Greenfield, Itzhaq Shai, Tina L. Greenfield, Elizabeth R. Arnold, Annie Brown, Adi Eliyahu-Behar, Aren M. Maeir

The sixth author’s name is spelled incorrectly. The correct name is: Adi Eliyahu-Behar. The correct citation is: Greenfield HJ, Shai I, Greenfield TL, Arnold ER, Brown A, Eliyahu-Behar A, et al. (2018) Earliest evidence for equid bit wear in the ancient Near East: The "ass" from Early Bronze Age Tell eṣ-Ṣâfi/Gath, Israel. PLoS ONE 13(5): e0196335. https://doi.org/10.1371/journal.pone.0196335
